# Physical Activity and Sedentary Behavior of Children in Afterschool Programs: An Accelerometer-Based Analysis in Full-Day and Half-Day Elementary Schools in Germany

**DOI:** 10.3389/fpubh.2020.00463

**Published:** 2020-09-02

**Authors:** Arvid Kuritz, Christoph Mall, Melina Schnitzius, Filip Mess

**Affiliations:** ^1^Department of Sport Science, University of Konstanz, Konstanz, Germany; ^2^Department of Sport and Health Sciences, Technical University of Munich, Munich, Germany

**Keywords:** physical activity, sedentary behavior, full-day school, after-school program, children, accelerometry

## Abstract

**Background:** Regular physical activity (PA) and reduced sedentary behavior (SB) are positively related to children's health and considered as pillars of a healthy lifestyle. Full-day schools with their afterschool programs (ASPs) have an impact on children's daily PA and SB. Studies investigating PA and SB in ASPs, which compare PA and SB between the organizational forms full-day and half-day schools, are rare. The aim of this study is to describe elementary school children's PA and SB during ASPs and to compare the results to other time periods of the day, e.g., teaching hours and leisure time. Additionally, PA and SB of children in full-day and half-day schools are compared. Further, relevant factors influencing the achievement of the World Health Organization's (WHO's) PA guidelines for children, e.g., time spent in ASPs, are investigated.

**Methods:** PA and SB of 332 German students (*n* = 198 full-day school children; *n* = 134 half-day school children) from 11 different elementary schools were measured via accelerometry for 5 consecutive days within one school week in 2017. PA and SB during ASPs and other times of the day were analyzed via one-way and factorial ANOVA, correlation, and logistic regression.

**Results:** Children attending full-day schools show the highest percentage of moderate-to-vigorous PA (MVPA) (13.7%) and the lowest percentage of SB (49.5%) during ASPs, in comparison with teaching hours and leisure time. In the afternoon hours, full-day school children show 20 min less SB than half-day school children. Children spending more time in ASPs obtain significantly more SB (*r* = 0.23) and less MVPA (*r* = −0.15). Further, they less likely reach WHO's PA guidelines odds ratio (OR = 0.98).

**Conclusion:** Peers and the choice as well as offer of extracurricular activities promote PA in ASPs. Media availability leads to higher SB in leisure time. ASPs help to be more active and less sedentary. Time spent in ASPs should be limited, so that full-day school children still have the possibility to join other PA offers in leisure time. ASP time should contain a certain minimum amount of MVPA in line with ASP guidelines.

## Introduction

Physical inactivity and sedentary behavior (SB) are determinants of poor health (1). On the contrary, a basic level of physical activity (PA) is one pillar of a healthy lifestyle. There is manifold evidence for health benefits resulting from regular PA (2, 3) and reduced SB (1). Especially for children, regular PA and reduced SB are important health factors. Based on Dahlgren and Whitehead's (4, 5) model of *Social Determinants of Health*, behavior patterns such as PA and SB are significantly influenced by the social network and living as well as working conditions. For children, these living and working conditions are primarily the family, the commune they grow up in, and the school they attend. Considering the amount of time children spend at school on weekdays, the factor *school* contributes substantially to children's daily PA as well as SB and therefore their health.

In industrialized countries, almost every child is enrolled in school, and school has the most intensive and continuous impact on children aged 6–15 (6). Overall, in Germany, two organizational forms of school exist: full-day schools and half-day schools. The main difference is the amount of time spent at school. Children at half-day schools on average stay 5 h in school. Children at full-day schools on average spend 7–8 h in school. Time beyond teaching hours is usually spent in extracurricular activities of afterschool programs (ASPs). This extra time in full-day schools also provides a large amount of free time for PA, and this can reduce SB. In Europe, most countries offer full-day school programs (7). In the USA, extracurricular activities are usually part of the school system. The organizational form of Germany's school system, especially considering elementary schools, currently changes from half-day to full-day schools. Full-day schools feature diverse characteristics: mandatory vs. voluntary attendance, partly rhythmization of lessons and ASPs as well as a varying overall duration. Parents can typically choose if their children attend a full-day or half-day class within one school. All full-day schools offer a variety of ASPs. Sports activities are the most frequent offers in German full-day school ASPs (8, 9). Federal regulations ensure that either teachers or trained staff organize and guide ASPs or observe free play. Children typically have to sign up for an activity for one term. Age groups are usually mixed. A standard school day in German full-day schools starts with teaching in the morning until lunch break, followed by rarely more teaching or time for homework and extracurricular activities in the afternoon. Prolonged attendance at full-day schools compared with half-day schools impacts children's lifestyle and leisure behavior as well as health behavior depending on the organizational form or ASPs' duration. Full-day schools providing PA offers within ASPs in a low-threshold way can help to reach guidelines for PA and SB, independent of social network (4, 5) or participation in organized sports (10). Full-day schools can therefore influence health-related behavior patterns.

The World Health Organization (WHO) recommends at least 60 min of moderate-to-vigorous PA (MVPA) daily for children aged 5–17 (11). These *Global Recommendations on Physical Activity for Health* (WHO PA GL) are a marker for a healthy lifestyle in the meaning of daily PA. Besides the WHO PA GL, there are several recommendations, policies, or guidelines regarding the amount of PA in ASPs (12). These requirements postulate that up to 60 min of MVPA should take place in ASPs (13–15). It is also recommended that 20–50% of PA in ASPs should be MVPA (14). Most organizations, e.g., the United States National Afterschool Association, call for 30 min MVPA in ASPs (14). This means that ASPs should provide half of the recommended MVPA (12). The WHO PA GL states physical inactivity or SB as evidence of poor health. Specific recommendations regarding SB for children aged 5–17 are missing. In Germany, *National Recommendations for Physical Activity and Physical Activity Promotion* (16) recommends as little as possible and a maximum of 60 min daily sitting, using a screen media or SB. Guidelines for ASPs also recommend a screen time of <60 min (13–15). Screen time should be limited to homework, research, or digital learning (14). The California Department of Education also recommends a maximum 60 min of SB in ASPs (13).

Several studies investigated PA and SB in the school context, especially considering full-day school's ASPs: Beets et al. (17) showed in their study with more than 1,000 elementary school children attending 97 ASPs in South Carolina, USA, operated by the Young Men's Christian Association (YMCA), that only one quarter reached the 30-min guideline of PA in ASPs. On average, children spent 21.4 min in MVPA and 64.3 min in SB at baseline. In the study, ASP staff qualification was also investigated with the assumption that trained staff can increase students' MVPA during ASPs. Regarding children's overall PA in combination with ASP's, PA helps to identify active periods within 1 day. De Meester et al. (10) investigated daily PA of 1,526 Belgian students and compared participants of extracurricular school-based sports offerings with non-participants via questionnaire. Participants were significantly more physically active than non-participants, even after controlling for outer school sports participation. They concluded that ASP participation is linked to higher PA levels. Therefore, full-day school may contribute to an active and healthy lifestyle. Pau et al. (18) measured PA and SB via accelerometry in 169 Italian elementary school children. They compared full-day and half-day school children's PA and SB in different time periods. In the afternoon time period, where ASPs take place for full-day attendants, children spent significantly less time in SB and more time in MVPA than did half-day school children spending the afternoon hours outside school. Van Stralen et al. (19) investigated in a cross-European cross-sectional survey (The ENERGY project) PA and SB during the school day. Ten- to 12-year-old European school children spent on average 65% of their time at school in SB and 5% in MVPA. Van Stralen et al. found significant differences in SB and MVPA for boys and girls. Boys showed less SB and higher amounts of MVPA than did girls. In Messing et al. (20) systematic review of reviews considering PA promotion among children and adolescents, few studies analyzed time in ASPs and identified these programs as effective health promoter. Regarding associations between PA and ASPs, especially programs focusing on PA and/or sports were categorized as effective. Other studies investigated PA and SB in students or in school, respectively, but without specific reference to full-day school or ASPs. Systematic reviews from Parrish et al. (21) or Atkin et al. (22) provided an overview of studies and results in this field. The analyzed studies either took place during recess or lunch break (21) or examined additionally implemented short-term interventions to promote PA and reduce SB in school (22). For this reason, comparisons with these findings are limited to the field of PA and SB in ASPs in full-day schools.

Overall, most studies in this field analyzed children's daily PA and SB, without differentiating between times of the day. Research analyzing PA and SB in ASPs and its potential influence on daily PA as well as studies comparing full-day school and half-day school are rudimentary. Studies investigating PA and SB in German full-day schools accurately and with device-based measurements, such as accelerometry, are missing. Only one international study (18) compared full-day and half-day school children's PA and SB via accelerometry. Considering this state of studies, questions about full-day schools' or particularly ASP's contribution to children's PA and SB cannot be answered clearly. Further, a comparison between PA and SB in half-day and full-day school children is still pending. Against this background, this study has three major aims:

1. the description of PA and SB of children attending full-day schools during time spent in ASPs, and additionally, the comparison of PA and SB in ASPs with PA and SB during teaching hours and leisure time;
2. the comparison of full-day school and half-day school children's PA and SB during the afternoon hours; and
3. the investigation of the impact of full-day school specific factors on the achievement of WHO PA GL.

## Materials and Methods

### Study Design

This study was part of the project *Physical Activity in Full-Day School Children* funded by the German Federal Ministry of Education, Youth and Sports Baden Württemberg from 2014 to 2018. The study was performed from May to July 2017 in elementary schools with either a mandatory or voluntary full-day school branch located in the federal state of Baden-Württemberg, Germany. To minimize weather condition-related differences in PA and SB, data were collected in spring and summer. Regional weather conditions (hours of sunshine, temperature, and daily rainfall) were documented and checked using data from the German Weather Service.

In the first step, 25 schools—officially registered, accredited, and therefore supported by the Federal Ministry of Education, Youth and Sports Baden-Württemberg in their first year as full-day school—were asked to join the study. Eleven schools (44%) agreed to participate. Overall, 1,620 children from grade 1 to 4 were enrolled in the 11 participating schools. In the second step, principals and class teachers handed out an information letter about the study. Parents were asked to fill out the included reply form to accept or decline their children's participation in the study. Regardless of their affiliation to half-day or full-day school, all children of grade 1 to grade 4 were invited to participate. Data were collected by accelerometry for device-based measurement of PA and SB and by paper–pencil questionnaire (23).

### Participants and Data Collection

**Figure 1:**
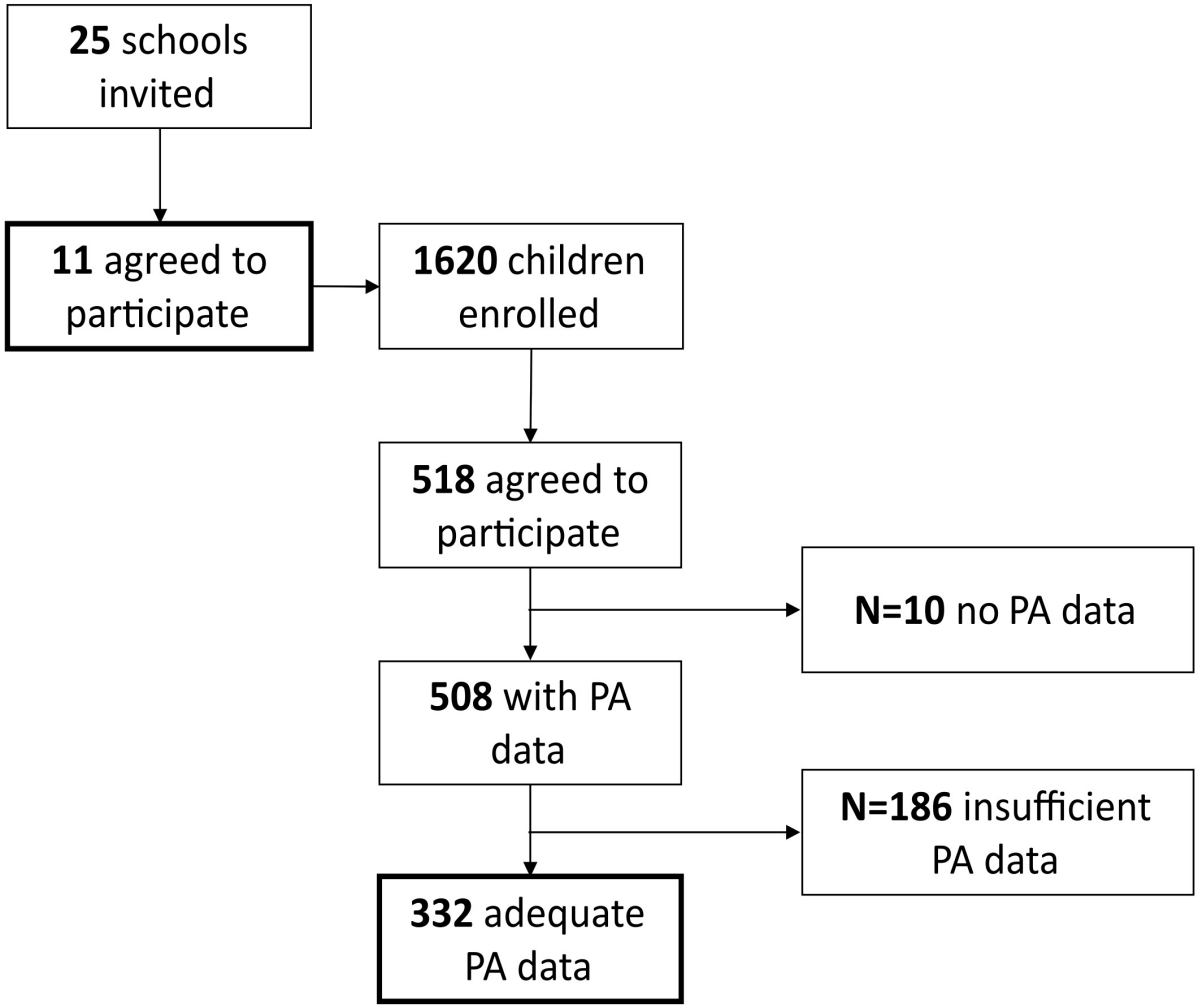
Flow chart of the sample selection process.

In total, 508 children were equipped with accelerometers and paper–pencil questionnaires. The test period was a regular week without special events (e.g., federal youth games, excursions, and sports meetings). Because of broken devices (*n* = 10) and lack of wear time (*n* = 186), the final sample included 332 children with adequate data for 5 consecutive school days (Figure 1). The reason to exclude students with insufficient data is based on the study of Rich et al. (24) determining minimum wear time for accelerometer data. A measurement reliability coefficient of 0.92 can be achieved by a wear time of at least 6 h/day on 5 days. Children's age ranged from 6.69 to 12.3 years with an average of 8.97 (SD = 1.2) years. Sex, organizational form of school, and grade level were well-distributed. Table 1 shows detailed sample characteristics.

**Table 1:** Sample and characteristics.

*N*	332
Age (mean, SD)	8.97 (±1.2)
Age (range)	6.69–12.3
Sex	
Female	172 (51.8%)
Male	160 (48.2%)
Organizational form	
Half-day school	134 (40.4%)
Full-day school	198 (59.6%)
Grade level	
1st	77 (23.2%)
2nd	88 (26.5%)
3rd	90 (27.1%)
4th	62 (18.7%)
Mixed-age classes	15 (4,5%)
Students per school	30.18 (±14.0)

### Measures

Participating children wore a tri-axial accelerometer (ActiGraph® GT3X, GT3X+ or, GT3XBT, Acticorp Co., Pensacola, USA) at the left hip with an elastic belt. Robusto and Trost's (25) study highlighted a strong agreement between the different models of applied ActiGraph accelerometer monitors. Children were asked to wear the accelerometer as soon as they got up in the morning and only remove it during water-based activities, at night and in exceptional cases because of the risk of injury, e.g., while performing contact sports such as martial arts.

Data collection by accelerometer was set to 10-s epoch length and started 1–2 h after distribution to avoid increased PA results because of curiosity for device and study. ActiLife Software v6.11.9 was used for data processing. Wear time validation was calculated using Troiano (26) defaults. Time intervals of at least 60 consecutive minutes of zero counts were defined as non-wear time. For PA level calculation and identification of SB, Pulsford's cut points for children (27) were used. Therefore, counts per minute of the vertical axis were classified as SB (SB < 100), light PA (LPA ≤ 2,240), moderate PA (MPA ≤ 3,840), and vigorous PA (VPA ≥ 3,841).

Socio-demographic data (date of birth and sex) were collected via paper–pencil questionnaire, which was an adapted version of the *Motorik-Modul activity questionnaire MoMo-AFB* (23). Children filled out the questionnaire together with their parents or guardians. In the questionnaire, parents and children were asked if the child attended full-day or half-day school. If attending full-day school, ASP end time or time leaving school should be specified for each day separately. Additional information about class and ASP schedules or other school-based information was given by the schools' principals or teaching staff.

### Data Preparation and Data Analyses

PA and SB data were divided into different time periods throughout the day and summarized for 5 consecutive school days from Monday to Friday. Individual timetables were created, based on class schedule and ASP attendance information. A typical school day was divided into teaching hours and leisure time for half-day school children and teaching hours, ASPs, and leisure time for full-day school children. To compare PA and SB in the afternoon hours between full-day and half-day school children, an additional time period named *non-teaching* time was created. Children attending full-day schools stay in school for extracurricular activities in ASPs. Leisure time with activities free of choice started after ASPs. *Non-teaching* time for full-day school children included ASPs and leisure time. Children in half-day schools left school after teaching hours. The remaining time awake was leisure time and therefore equal to *non-teaching* time. Valid wear time was checked for full day recording (as mentioned above) and double-checked for each period of the day to ensure at least 50% wear time in each time period. On average, daily wear time amounted to 11.92 (SD = 2.3) hours and 49.7% (SD = 9.5) for a whole day; 4.06 (SD = 0.7) hours in teaching hours, which equates to 89.3% (SD = 12.8) for daily teaching hours, 2.46 (SD = 1.0) hours and 91.6% (SD = 14.4) in ASPs, 6.72 (SD = 2.1) hours and 50.6% (SD = 13.5) in leisure time, and 7.75 (SD = 1.9) hours and 54.1% (SD = 12.5) in *non-teaching* time.

After raw data to PA and SB data with ActiLife® software were converted, Microsoft® Excel® for Office 365 MSO was used to prepare data.

Comparisons of children's PA and SB in different time periods (e.g., ASPs and leisure time) within the day were conducted by repeated measure factorial ANOVA with sex and grade as independent variables. Analyses comparing full-day and half-day school children or subgroups differentiated by school affiliation were fulfilled by one-way ANOVA, to identify the interaction effect between sex and grade, a factorial ANOVA was conducted. For multiple comparisons, Tukey's *post-hoc* test was used. Effect size eta squared was categorized based on Cohen (28): small, η^2^ < 0.06; medium, η^2^ = 0.06 to 0.14; and large, η^2^ > 0.14. If sphericity was missing, Greenhouse–Geisser correction was applied. Correlations between variables were calculated via Pearson correlation coefficient. Prediction for school's organizational form attendance and WHO PA GL achievement was checked with chi-squared test for the whole sample. The subsample of full-day school children was analyzed by logistic regression to identify general (sex and age) and specific full-day school predictors (number of days per week in ASPs, duration of ASPs in minutes per day, minutes of MVPA in ASPs per day, and reaching the guideline of 20% MVPA in ASPs) as independent variables, which were given as odds ratio (OR) to achieve the WHO PA GL (dependent variable). Significance was assessed at α = 0.05 for all analyses. All statistical tests were conducted using IBM® SPSS® Statistics (version 26).

## Results

### Children's Physical Activity and Sedentary Behavior in After-School Programs

The following results refer to the subgroup of participants attending full-day school (*n* = 198) and taking part in ASPs on at least 1 and up to 5 days of the week. Over two thirds of students attending full-day school participate on three (31.8%) or four (37.4%) afternoons in ASPs. The number of days varies in the full range from one (6.6%) or two (7.1%), up to five (17.2%) days attending full-day school programs within a school week with an average of 3.52 (SD = 1.1) days weekly. On average, children spend 162.88 (SD = 57.0) minutes per day in ASPs. The maximum duration of ASPs time is 5 h/day. Summarizing all school days within 1 week, we found that children attend extracurricular activities in ASPs for 9 h 42 min (M = 582.45 min, SD = 285.7) on average.

**Figure 2:**
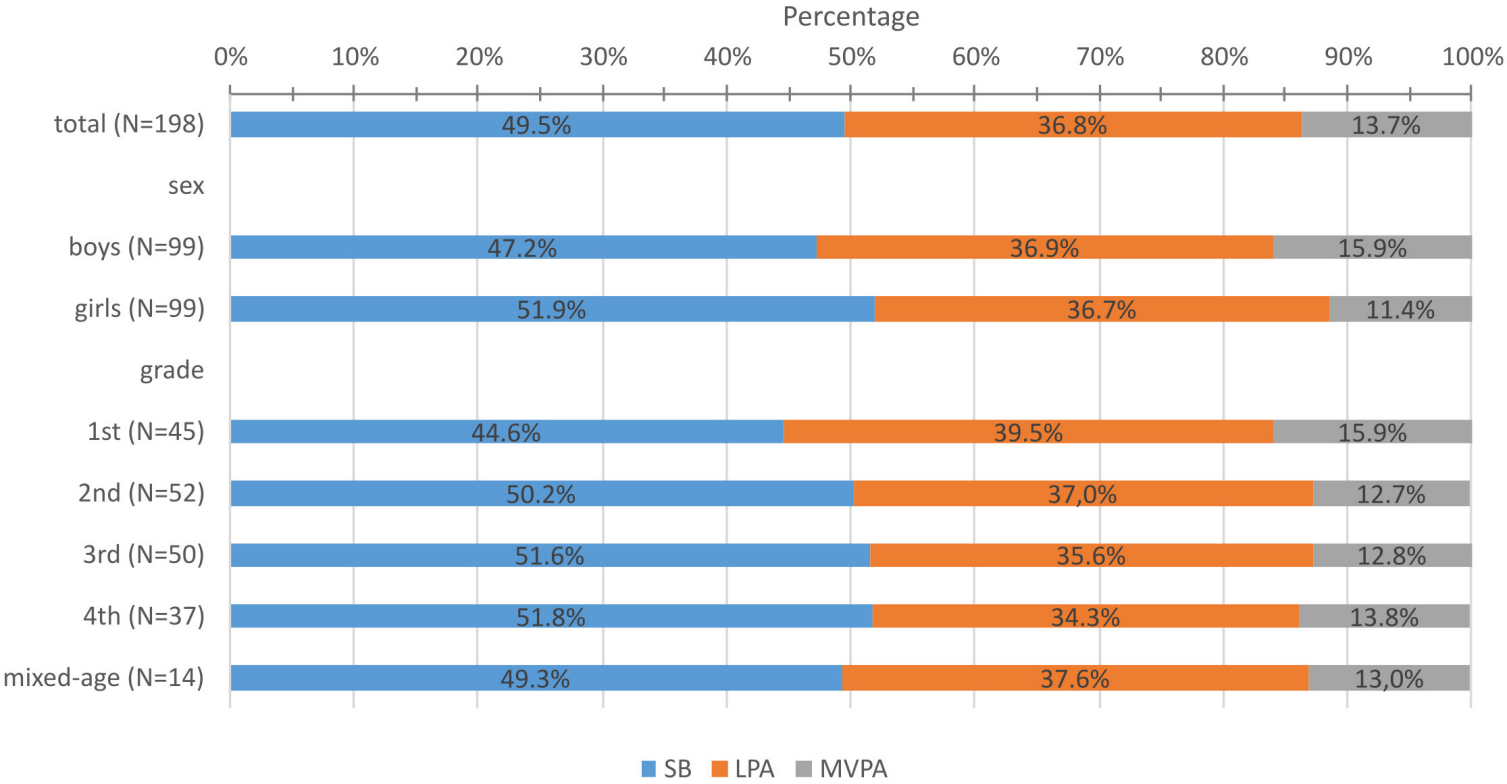
Percentages of physical activity and sedentary behavior in after-school program time of full-day school children.

Figure 2 shows the constitution of SB, LPA, and MVPA in ASPs for the full-day school subgroup—differentiated by sex and grade (and therefore indirectly by age). In ASPs, factorial ANOVA reveals no significant interaction for grade and sex [*F*_(4,188)_ = 1.32, *p* = 0.263, η^2^ = 0.03] in percentage of SB. Both factors individually show significant differences: boys' SB is lower than girls' [*F*_(1,198)_ = 8.34, *p* < 0.001, η^2^ = 0.04]. Differentiated by grade, there is a significant difference of SB in ASPs [*F*_(4,198)_ = 4.24, *p* = 0.003, η^2^ = 0.08]. Tukey's *post-hoc* multiple comparisons show that only first graders show significantly less SB than do second, third, and fourth graders. Considering the percentage of time spent in MVPA during ASPs, only sex shows a significant difference [*F*_(1,198)_ = 28.19, *p* < 0.001, η^2^ = 0.13]. There are neither significant differences between grades [*F*_(4,198)_ = 1.99, *p* = 0.107, η^2^ = 0.04] nor an interaction effect [*F*_(4,188)_ = 0.65, *p* = 0.625, η^2^ = 0.01]. Significant differences with large effects are found for school affiliation in both percentage in SB [*F*_(10,198)_ = 5.42, *p* < 0.001, η^2^ = 0.23] and MVPA [*F*_(10,198)_ = 3.53, *p* < 0.001, η^2^ = 0.16]. *Post-hoc* results reveal that only one school stands out with a highly active ASP. This school is the only one in the sample where full-day school is mandatory for all children. PA and SB data of this school (*n* = 27) show that children spend on average 18.4% (SD = 8.2) of ASPs time in MVPA (overall: M = 13.7%, SD = 6.2), which differs significantly in comparison with four schools in a multiple comparison. In 39.0% (SD = 10.0) of ASP time, children of this school are sedentary (overall: M = 49.5%, SD = 9.9), which is significantly different to eight schools.

**Figure 3:**
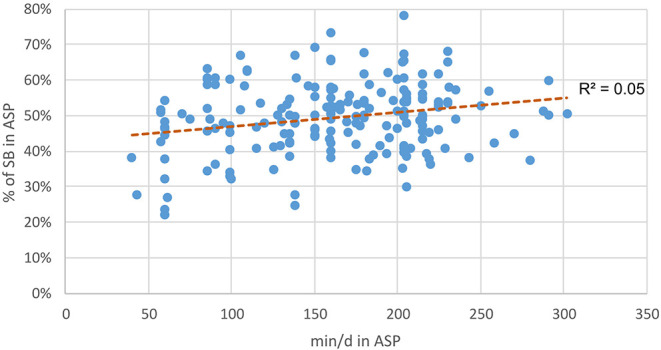
Duration of after school program (ASP) correlated to percentage of time in sedentary behavior in ASP.

**Figure 4:**
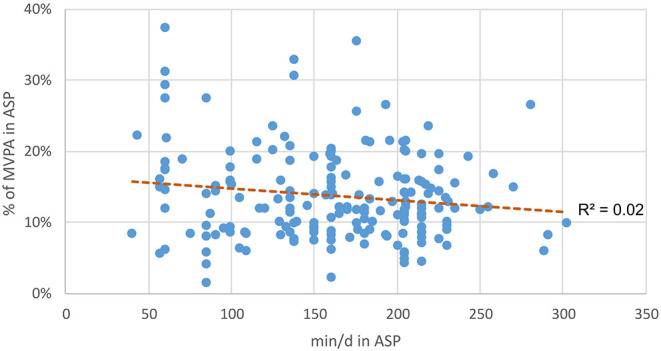
Duration of after school program (ASP) correlated to percentage of time in moderate-to-vigorous physical activity (MVPA) in ASP.

Children attending full-day school stay on average 2 h 43 min (M = 162.88 min, SD = 57.0) per school day at school for extracurricular activities in ASPs. Regarding the duration, there is a small positive correlation for SB (*r* = 0.23, *p* = 0.001) and a slightly negative correlation for MVPA percentages (*r* = −0.15, *p* = 0.037) as seen in Figures 3, 4. This shows that children who spend more minutes per day in ASPs show a higher percentage of SB and a lower percentage of MVPA than do children who spend less time in ASPs.

For children attending full-day school, ASP (SB: M = 49.5%, SD = 9.9; MVPA: M = 13.7%, SD = 6.2) time is significantly the most active and least sedentary time of the day than are teaching hours (SB: M = 61.6%, SD = 7.0; MVPA: M = 8.4%, SD = 2.9) and leisure time (SB: M = 57.9%, SD = 7.6; MVPA: M = 10.1%, SD = 3.5) with the highest percentage of MVPA and lowest percentage of SB. Considering SB, interaction effects of the repeated measure factorial ANOVA are significant for the time periods differentiated by sex, but not by grade or by sex and grade. For percentages of MVPA, significant interaction effects can be found for sex as well as grade, but not for grade and sex (see Tables 2, 3). Descriptive data can be found in Supplementary Tables A, B.

**Table 2:** Differences of percentages in time spent in sedentary behavior (SB) in different periods of the day in full-day school children (*n* = 198).

	**Test for difference**	***p***	***η^2^***
Setting^a^	*F*_(1.87,351.47)_ = 138.86	<0.001	0.43
Setting × grade^a^	*F*_(7.48,351.47)_ = 1.89	0.066	0.04
Setting × sex^a^	*F*_(1.87,351.47)_ = 4.03	0.021	0.02
Setting × grade × sex^a^	*F*_(7.48,351.47)_ = 1.43	0.189	0.03

a *Sphericity not given, Greenhouse–Geisser correction*.

**Table 3:** Differences of percentages in time spent in moderate to vigorous physical activity (MVPA) in different periods of the day in full-day school children (*n* = 198).

	***F***	***p***	***η^2^***
Setting^a^	*F*_(1.66,312.60)_ = 83.94	<0.001	0.31
Setting × grade^a^	*F*_(6.65,312.60)_ = 2.38	0.024	0.05
Setting × sex^a^	*F*_(1.66,312.60)_ = 8.41	0.001	0.04
Setting × grade × sex^a^	*F*_(6.65,312.60)_ = 0.70	0.667	0.02

a *Sphericity not given, Greenhouse–Geisser correction*.

### Comparison of Full-Day and Half-Day School Children's Physical Activity and Sedentary Behavior in the Afternoon Hours

During *non-teaching* time children attending full-day schools (M = 256.84 min, SD = 76.45) spend significantly less minutes in SB than children in half-day schools (M = 276.60 min, SD = 89.0): *F*_(1,332)_ = 4.67, *p* = 0.031, η^2^ = 0.01. The difference in minutes of MVPA in the *non-teaching* time is not significant; half-day school children (M = 47.57 min, SD = 19.59) spend 1.7 min less in MVPA than children attending full-day schools (M = 49.26 min, SD = 18.60): *F*_(1,332)_ = 0.63, *p* = 0.427, η^2^ < 0.01. Considering girls and boys separately, the affiliation to school's organizational forms do not differ in boys' SB [*F*_(1,160)_ = 0.33, *p* = 0.570, η^2^ < 0.01) or MVPA [*F*_(1,160)_ = 0.33, *p* = 0.565, η^2^ < 0.01). The difference in minutes of SB in the *non-teaching* time for girls aggregates to almost 30 min. Girls in half-day schools show SB for 290.47 (SD = 87.19) minutes and girls in full-day schools for 260.73 (SD = 85.29) min [*F*_(1,172)_ = 5.02, *p* = 0.026, η^2^ = 0.03). As reported for boys' PA, there are no significant differences in girls' MVPA [*F*_(1,172)_ = 0.06, *p* = 0.801, η^2^ < 0.01). Regarding subgroups divided by grade, there is a difference in second graders: half-day school children in second grade spend significantly more time in SB during *non-teaching* time than full-day school children [*F*_(1,88)_ = 5.75, *p* = 0.019, η^2^ = 0.06). For all other grades, differences in SB and PA between full-day and half-day school children are not significant.

### The Global Recommendations on Physical Activity for Health: The Influence of Specific Full-Day School and General Variables

In this study, 59.9% of participants reach the recommended 60 min of MVPA daily according to the WHO PA GL (11). A chi-squared test shows that significantly more boys (73.8%) than girls (47.1%) can be categorized as active in the meaning of the WHO (χ^2^(1) = 24.53, *p* < 0.001, φ = 0.27). Attendance to full-day or half-day school does not show any significant difference in the percentage of children reaching the WHO PA GL: full-day school 62.1%, half-day school 56.7% (χ^2^(1) = 0.97, *p* = 0.324, φ = 0.05).

The logistic regression model with six predictors reaches a likelihood of 79.1%. The OR is displayed in Table 4. Sex as a significant predictor in this model shows that boys are more than twice as much likely (OR = 2.16) than girls to reach the WHO PA GL. Age and number of days per week attending ASPs do not show any significant predictions. The likelihood to reach the WHO PA GL decreases (OR = 0.98) when duration of ASPs increases. Vice versa, an increased number of minutes of MVPA in ASPs helps to increase the likelihood of reaching the guideline (OR = 1.26). Reaching the ASP guideline of 20% MVPA of total time in ASPs does not show a significant influence on the dependent variable.

**Table 4:** Logistic regression and odds ratio (OR) for reaching the Global Recommendations on Physical Activity for Health (WHO PA GL).

**Variable**	**OR**	**95% CI**		***p*-value**
		**Low**	**High**	
Sex (ref. girl)	ref.			0.048
Boy	2.16	1.01	4.63	
Age	0.94	0.67	1.31	0.696
Number of days per week in ASP (ref. 1 day)	ref.			0.605
2 days	4.18	0.50	35.19	0.188
3 days	2.05	0.39	10.73	0.394
4 days	2.51	0.47	13.44	0.281
5 days	1.46	0.24	8.83	0.684
Duration of ASP (min/day)	0.98	0.97	0.99	<0.001
Minutes of MVPA in ASP (min/day)	1.26	1.16	1.36	<0.001
Reaching the guideline of 20% MVPA in ASP	0.39	0.08	2.01	0.259

*Total assumption: χ^2^(9) = 84.91, p < 0.001. Effect size: Nagelkerke's R^2^ = 0.48*.

## Discussion

### After-School Program as Highly Active Time of the Day

Germany's change from the traditional half-day to a full-day school system may bring along changes in children's lifestyle, leisure time, and PA as well as SB habits. Hence, the first aim of this study was to describe and analyze PA and SB in ASPs of children attending full-day schools and compare the results considering ASPs with other times of the day. Girls as well as children in higher grades show more SB and less MVPA in ASPs than boys or children in lower grades, respectively. These well-known differences (29) may lead to a policy of organizing ASPs in full-day schools: it is essential to offer various activities in ASPs to address all children independent of sex or age. In elementary schools, age-appropriate activities, specifically for older children in grades 3 and 4, may motivate this age group to be physically active and by this decrease their SB time. Additionally, results showed that ASP time is characterized by high percentages of MVPA as well as low percentages of SB. ASPs therefore depict the most active time of the day in comparison with teaching hours or leisure time. Three points may explain this highly active time: first, at German full-day schools, most ASP activities involve sports, games, or play. Almost every school provides at least one PA-based offer within its full-day school program (8, 9). Second, children attending ASPs have access to facilities that allow or promote PA, e.g., schoolyards. Access to facilities promotes PA and therefore reduces SB (30, 31). In the case of full-day schools, availability and access to the schoolyard or in some cases the school's own gym may help to encourage children for PA (32). Third, leisure time at home is often characterized by SB in the form of screen usage (33). At school, screen usage is restricted to educational purposes, which the *National Afterschool Association* (14) also recommends for ASPs. Time for highly active offers in ASPs seem to be limited. Prolonged attendance in ASP correlates with a higher percentage of time spent in SB. One reason might be full-day schools' organization. In this sample, federal requirements ask for 7–8 h in school on 3–4 days. Further, many full-day schools offer privately organized and fee-based voluntary childcare exceeding after-school hours. In this time beyond the federal required ASPs, less children are present. Consequently, there is less variety of organized activities, and the possibility to play freely with other children that brings along PA decreases. The lack of peers or offers therefore may be reason for the higher percentage of SB in prolonged ASPs. Furthermore, there may be an infrastructural reason for less PA and more SB in prolonged ASPs: schools and sports clubs typically share facilities. As sports clubs' activities take place in the later afternoon hours, the availability of gyms and playing fields (e.g., soccer pitches or athletics fields) for activities in ASPs is limited. This double usage of sports facilities is a limiting factor for extracurricular activities in ASPs (34) in general and in prolonged ASPs in particular. Because of these reasons, it can be assumed that staying in full-day schools for an extended time may involve an unorganized and SB-based waiting for pickup by parents or guardians.

### Comparison of Full-Day and Half-Day School Children's Physical Activity and Sedentary Behavior

The second major aim of this study was to compare PA as well as SB of children in full-day and half-day schools during the afternoon hours in the *non-teaching* time period. Children attending full-day schools showed less SB than children in half-day schools in the afternoon hours. For the same time period, this study did not find significant differences in MVPA. The results can confirm Pau et al. (18) results regarding lower SB, but not regarding higher MVPA among full-day school children in comparison with half-day school children. According to Dahlgren and Whitehead's (4, 5) model, living conditions and the social network are different in full-day and half-day school children's *non-teaching* time. On the one hand, in ASPs, peers and space for free play or organized extracurricular activities are available. This enables full-day school children to spend their time less sedentary and more active in ASPs as compared with half-day school children who potentially spend the early afternoon at home. On the other hand, referring data of this study, Spengler et al. (35) showed that full-day school children are less engaged in organized sports outside school than are half-day school children. Furthermore, weekly duration of training in sports clubs is significantly higher in half-day school children compared with full-day school children. This may explain that there is no significant difference in MVPA between children attending full-day and half-day schools in the afternoon hours. Against this background, it seems that Züchner and Arnoldt's (36) hypothesis considering the changes in children's sports club activities in connection with full-day school attendance can be accepted: a shift of PA in leisure time from sports club activities to ASPs for full-day school children seems possible. ASPs may be a chance to increase MVPA and decrease SB of children who do not participate in organized sports activities in their leisure time (10). With the aim to reduce SB and increase PA, three policies seem promising: first, half-day school children could join ASPs to reduce their SB in the early afternoon, independent of participation in organized sports. Second, children attending ASPs in full-day schools should be motivated to participate in club sports or to join free play in the late afternoon. And third, schools should provide an obligatory PA time during ASPs but in accordance to a designated duration of ASPs. Therefore, a guideline for PA and SB in ASPs should include both absolute and relative values as recommendations for PA and SB. This finding can also decline critics' assumption that full-day school will only increase SB.

### After-School Program in Full-Day Schools and the Global Recommendations on Physical Activity for Health

The third aim of this study was to identify potential and specific full-day school factors that influence the achievement of WHO PA GL. The schools' organizational form did not influence reaching the boundary value of 60 min MVPA daily. Analyzing predictors identified gender in favor of boys, shorter duration of ASPs, and higher number of minutes of MVPA in ASPs as beneficial to reach WHO PA GL. The gender effect in favor of boys is comparable with that of the study of Beets et al. (29). Combining these findings, quantity and quality of ASPs are relevant for children's PA and should be adjusted. This means that beyond the obligatory lunch (federal requirements) and time for homework, ASPs should provide time to be active in extracurricular activities. Prolonged attendance without activities may lead to sedentariness and by this decreases the possibility to reach WHO PA GL.

### Future Research and Practical Implications

Future research should investigate children's PA and SB longitudinally. PA and SB development or changes over time comparing different organizational forms could be displayed. Transitions from half-day to full-day school or vice versa may bring along changes in lifestyle habits, sports club participation, and other factors influencing daily PA and SB. This study's results considering PA and SB only show children's behavior in spring and summer season with mostly good weather conditions. Comparisons between other seasons of the year or the influence of weather conditions for full-day and half-day school children's PA and SB would be interesting, too. This study analyzed device-based measured PA and SB via accelerometry. ASP content was not observed. To identify offers in ASPs that lead to higher or lower PA, ASP content should be analyzed in combination with PA and SB data. As research in physical education classes explains quality of teaching content and staff qualification, these aspects should be investigated in ASPs likewise. Examining the optimal duration and content of ASPs benefitting children's daily PA and SB requires multiple findings regarding the abovementioned topics. Including children's and parents' or guardians' socioeconomic status may (a) help to identify specific subgroups or (b) highlight the school's organizational form influence on children's PA and SB. Compared with sports clubs, full-day schools with optional sports activities reach more children. A potential influence of socioeconomic issues on children's sports activities may be much smaller in full-day schools' ASPs than in organized activities in sports clubs. Additionally, activities in ASPs could have a recruitment effect on club sports (36).

Findings of this study may lead to the following practical implications: time in ASPs should be limited in duration and not exceed the late afternoon. Full-day schools should offer a variety of extracurricular activities in ASPs for the time children stay in school. Children attending full-day school should still have the possibility to participate in organized sports, e.g., organized by sports clubs. Therefore, full-day schools and sports clubs should cooperate. Because of a lack of teachers, providing extracurricular activities for the whole time in ASPs is a problem for many schools. Including sports club coaches in ASPs may help to offer more activities during ASPs and at the same time enable sports clubs' recruitment of interested children to participate in organized sports. The compliance of PA-based ASP guidelines may help to lead children not engaged in organized sports to more PA and less SB.

### Strengths and Limitations

A strength of this study is the device-based measurement of PA and SB via accelerometry with a sample size of *N* = 332 distributed over 11 elementary schools. Accelerometer-based PA and SB data underlies the chosen algorithms to calculate wear time and cut points. Algorithms used in this study were chosen by best fitting arguments. Comparisons with studies using other algorithms should be done carefully (37). Although, Robusto and Trost (25) as well as the producer postulate that the ActiGraph® models used in this study are completely compatible, a doubtless comparison of data seems to be only possible using totally identical device models. This study was conducted in the federal state of Baden-Württemberg, Germany. Therefore, the sample is not representative and is subjected to regional conditions. Schools' participation was voluntary. It can be assumed that schools declined their participation because of bad circumstances, e.g., schoolyards under construction, shortage of teachers, or the apprehension to achieve bad results and to be marked as *low PA school*. Further, children's participation was voluntary. The relatively low participation rate (31.8%) may be attributed to the considerable effort for parents, managing questionnaire completion, and accelerometer recording for a whole week. Although parents were informed about privacy and the Federal Ministry's approval of the study, data collection via accelerometry might still be seen critically by some parents.

## Data Availability Statement

The raw data supporting the conclusions of this article will be made available by the authors, without undue reservation.

## Ethics Statement

The studies involving human participants were reviewed and approved by Ethics Commitee of the University of Konstanz (approval number: 08/2019). Written informed consent to participate in this study was provided by the participants' legal guardian/next of kin.

## Author Contributions

AK and FM coordinated the whole project and developed the study design. AK collected data and wrote the paper with substantial contributions from CM and MS. AK and CM performed the data preparation and statistical analysis. MS performed the final proofreading. All authors provided feedback on drafts and approved the final manuscript.

## Conflict of Interest

The authors declare that the research was conducted in the absence of any commercial or financial relationships that could be construed as a potential conflict of interest.

## Abbreviations

PA: physical activity
SB: sedentary behavior
WHO: World Health Organization
ASP: after-school program
WHO PA GL: Global Recommendations on Physical Activity for Health
MVPA: moderate-to-vigorous physical activity
LPA: light physical activity
OR: odds ratio.
