# Supplementary material for: Physical Activity and Sedentary Behavior of Children in Afterschool Programs: An Accelerometer-Based Analysis in Full-Day and Half-Day Elementary Schools in Germany
Source: Front Public Health. 2020 Sep 2;8:463. doi: 10.3389/fpubh.2020.00463 (PMC7492590; doi:10.3389/fpubh.2020.00463)
Supplement: Supplementary file 1 [file Table_1.DOCX]

Physical Activity and Sedentary Behavior of Children in After School Programs: An Accelerometer-based Analysis in Full-day and Half-day Elementary Schools in Germany

Supplementary Material

Table A.Wear Time for Whole Day and the Time Periods Teaching Time, ASP Time, Leisure Time and Non-Teaching Time.

| Valid wear time | School week (Mon-Fri) whole day | teaching hours | ASP time (n = 198) | Leisure time | Non-teaching time |
| --- | --- | --- | --- | --- | --- |
| Percentage (Mean, SD) | 49.7% (±9.5) | 89.3% (±12.8) | 91.6% (±14.4) | 50.6% (±13.5) | 54.1% (12.5) |
| Hours per Day (Mean, SD) | 11.92 (±2.3) | 4.06 (±0.7) | 2.46 (±1.0) | 6.72 (±2.1) | 7.75 (±1.9) |

Table B. Descriptives of Percentages in Time Spend in SB in Different Periods of the Day in Full-Day School Children

|  | | percentage SB | | | | | | | | | percentage MVPA | | | | | | | | |
| --- | --- | --- | --- | --- | --- | --- | --- | --- | --- | --- | --- | --- | --- | --- | --- | --- | --- | --- | --- |
|  | N | teaching hours | | | ASP | | | leisure time | | | teaching hours | | | ASP | | | leisure time | | |
| overall* | 198 | 61.6 | ± | 7.0 | 49.5 | ± | 9.9 | 57.9 | ± | 7.6 | 8.4 | ± | 2.9 | 13.7 | ± | 6.2 | 10.1 | ± | 3.5 |
| Sex |  |  |  |  |  |  |  |  |  |  |  |  |  |  |  |  |  |  |  |
| boys* | 99 | 60.1 | ± | 6.9 | 47.2 | ± | 10.4 | 57.5 | ± | 7.8 | 9.5 | ± | 3.0 | 15.9 | ± | 6.6 | 11.0 | ± | 3.8 |
| girls* | 99 | 63.1 | ± | 6.9 | 51.9 | ± | 8.7 | 58.2 | ± | 7.5 | 7.3 | ± | 2.4 | 11.4 | ± | 4.8 | 9.2 | ± | 2.9 |
| grade |  |  |  |  |  |  |  |  |  |  |  |  |  |  |  |  |  |  |  |
| 1st* | 45 | 59.2 | ± | 5.7 | 44.6 | ± | 8.0 | 56.1 | ± | 6.9 | 8.6 | ± | 2.5 | 15.9 | ± | 5.6 | 10.5 | ± | 3.4 |
| 2nd* | 52 | 61.3 | ± | 6.2 | 50.2 | ± | 9.6 | 56.0 | ± | 7.8 | 8.4 | ± | 2.2 | 12.7 | ± | 4.9 | 10.3 | ± | 3.8 |
| 3rd* | 50 | 63.8 | ± | 6.0 | 51.6 | ± | 9.3 | 60.4 | ± | 7.4 | 7.7 | ± | 2.9 | 12.8 | ± | 6.6 | 9.3 | ± | 3.1 |
| 4th* | 37 | 64.1 | ± | 8.3 | 51.8 | ± | 11.4 | 60.4 | ± | 7.2 | 8.2 | ± | 3.1 | 13.8 | ± | 7.2 | 9.5 | ± | 3.4 |
| mixed-age* | 14 | 56.0 | ± | 8.3 | 49.3 | ± | 9.0 | 54.5 | ± | 6.9 | 10.7 | ± | 4.6 | 13.0 | ± | 6.8 | 12.8 | ± | 3.3 |
| *sig. differance between settings | | | | | | | | | | | | | | | | | | | |
